# Effectiveness of crisis resolution home treatment for the management of acute psychiatric crises in Southern Switzerland: a natural experiment based on geography

**DOI:** 10.1186/s12888-022-04020-z

**Published:** 2022-06-17

**Authors:** Emiliano Soldini, Maddalena Alippi, Maria Caiata Zufferey, Angela Lisi, Mario Lucchini, Emiliano Albanese, Raffaella Ada Colombo, Simona Rossa, Emilio Bolla, Zefiro Benedetto Mellacqua, Giuseppina Larghi, Severino Cordasco, Wolfram Kawohl, Luca Crivelli, Rafael Traber

**Affiliations:** 1 Department of Business Economics, Health and Social Care, University of Applied Sciences and Arts of Southern Switzerland, Palazzo E, Via Cantonale 16e, CH-6928 Manno, Switzerland; 2 Cantonal Psychiatric Clinic, Organizzazione Sociopsichiatrica Cantonale, Via Agostino Maspoli 6, CH-6850 Mendrisio, Switzerland; 3 Department of Sociology and Social Research, Università Degli Studi Di Milano Bicocca, piazza dell’Ateneo nuovo 1, 20126 Milan, Italy; 4 Institute of Public Health (IPH), Faculty of Biomedical Sciences, Università Della Svizzera Italiana, Via Giuseppe Buffi 13, CH-6900 Lugano, Switzerland; 5 Clienia Schlössli, Psychiatric Hospital, Schlösslistrasse 8, 8618 Oetwil Am See, Switzerland

**Keywords:** Acute psychiatric crises, Crisis Resolution Home Treatment, Inpatient treatment, Effectiveness, Quasi-experimental design

## Abstract

**Background:**

Crisis Resolution Home Treatment (CRHT) is an alternative to inpatient treatment for acute psychiatric crises management. However, evidence on CRHT effectiveness is still limited. In the Canton of Ticino (Southern Switzerland), in 2016 the regional public psychiatric hospital replaced one acute ward with a CRHT. The current study was designed within this evaluation setting to assess the effectiveness of CRHT compared to standard inpatient treatment.

**Methods:**

CRHT was offered to patients aged 18 to 65 with an acute psychiatric crisis that would have required hospitalization. We used a natural experiment based on geography, where intervention and control groups were formed according to the place of residence. Primary endpoints were reduction of psychiatric symptoms at discharge measured using the Health of the Nation Outcome Scales, treatment duration in days, and rate and length of readmissions during a two-year follow-up period after discharge. Safety during the treatment period was measured with the number of serious adverse events (suicide/suicide attempts, major self-harm episodes, acute alcohol/drug intoxications, aggressions to caregivers or family members). We used linear, log-linear and logistic regression models with propensity scores for the main analysis.

**Results:**

We enrolled 321 patients; 67 were excluded because the treatment period was too short and 17 because they were transferred before the end of the treatment. Two hundred thirty-seven patients were available for data analysis, 93 in the intervention group and 144 in the control group. No serious adverse event was observed during the treatment period in both groups. Reduction of psychiatric symptoms at discharge (*p*-value = 0.359), readmission rates (*p*-value = 0.563) and length of readmissions (*p*-value = 0.770) during the two-year follow-up period did not differ significantly between the two groups. Treatment duration was significantly higher in the treatment group (+ 29.6% on average, *p*-value = 0.002).

**Conclusions:**

CRHT was comparable to standard hospitalization in terms of psychiatric symptoms reduction, readmission rates and length of readmissions, but it was also characterized by a longer first treatment period. However, observational evidence following the study indicated that CRHT duration constantly lowered over time since its introduction in 2016 and became comparable to hospitalization, showing therefore to be an effective alternative also in terms of treatment length.

**Trial registration:**

ISRCTN38472626 (17/11/2020, retrospectively registered).

## Background

The worldwide growing emphasis on the socio-psychiatric approach in mental healthcare has contributed to the development of a variety of community-based services, which may help to reduce hospitalizations in psychiatric hospitals and clinics and to reorient mental health systems and services towards more community-based, patient-centred, integrated care [1–3]. Crisis Resolution Home Treatment (CRHT) provides an alternative setting to psychiatric hospitals for the treatment and care of acute psychiatric crises [4–6]. CRHT has gained a growing consensus over time because of its potential to leverage the psychosocial dimension intrinsically related to mental health crises [5] and preserve social participation and integration of people suffering from acute psychiatric crises, while also reducing the stigma associated with institutionalisation in traditional psychiatric settings [7, 8].

During the last 60 years, several CRHT experiences have been evaluated worldwide using a variety of study designs, including randomised controlled trials (RCT) [9–21], non-randomised comparative [22–26], and non-comparative [27–30] studies. Evidence synthesis endeavours are reported in systematic reviews [5, 31, 32], and highlight that CRHT can be an alternative to hospitalisation appreciated by patients and families. Ample evidence suggests that CRHT can be useful to foster the availability, accessibility, acceptability and integration of mental health services, as well as a multisectoral approach to attain a continuous and holistic care centred on needs of patients and their families. Next, observational evidence suggests that CRHT can contribute to reduce hospital admission/readmission rates and treatment length [33, 34]. However, evidence on the effectiveness of CRHT in the treatment of acute psychiatric crises is erratic because of the marked heterogeneity regarding the patients and the outcomes considered across studies. While many studies included patients suffering from an acute psychiatric crisis and requiring an immediate treatment, some included also patients without an immediate need of hospitalization [7, 19] or focused on the specific subgroup of schizophrenic patients [9, 15]. Moreover, patients with aggressive behaviour and/or self-arm and suicidality risk were sometimes well represented [17], but were excluded from other studies [20, 26]. Outcomes were also variable across studies. Hospital admissions, treatment length and/or readmission rates were the most widespread outcomes (included in nearly all studies), followed by clinical outcomes (e.g. psychiatric symptoms variation) also considered in many contributions [12, 15–17, 19, 20, 22, 24, 26]. Social outcomes (e.g. social inclusion or employment rates) were instead less frequently used [15, 17, 21, 24, 26], as well as patients’ and family members’ satisfaction with care [14, 16, 17, 20, 21, 23, 24] and family/relatives’ psychosocial burden [12, 14, 15, 22, 23]. The combination of the heterogeneity regarding patients and outcomes makes it difficult to have a robust comparison across studies, also because CRHT is a complex intervention and many other aspects (e.g. the aim of CRHT, the team composition or the geographical area served) may also have a great impact on its effectiveness. Moreover, some of the most recent studies were not useful for assessing the effectiveness of CRHT in comparison with standard inpatient treatment because they did not include the control group of hospitalized patients [27–30]. Therefore, evidence in favour of the effectiveness of CRHT as a substitute of hospitalization for acute crises treatment in current mental health systems remains limited.

From 2001 onwards, many different experiences of psychiatric home treatment have been implemented in Switzerland. The Cantons of Vaud [35–37], Thurgau and Basel introduced home treatment services after hospital discharge, but not as a replacement of hospital treatment and only for some patients, typically heavy users. CRHT teams that entirely replace an inpatient treatment have been implemented in the Cantons of Luzern [38], Aargau [20], Zürich [26] and Ticino, the Italian speaking region of the Swiss Confederation bordering northern Italy. In the latter case, a fully operational ward of the regional public psychiatric hospital (Cantonal Psychiatric Clinic, CPC) was shut down, and the staff of the ward was trained to build the CRHT team with the intention to substitute inpatient treatment.

The aim of the present study was to formally evaluate the effectiveness of the CRHT unit in Ticino as a substitute of standard in-hospital treatment. The main outcome measures were selected in order to ensure a good degree of comparability with the other two recent Swiss studies conducted in the cantons of Zürich [26] and Aargau [20], with the aim of providing additional external validity to the results collected in the Swiss context. These outcomes were reduction of psychiatric symptoms at the end of the treatment, treatment duration in days and rate and length of readmissions during a two-year follow-up period after discharge.

## Methods

The study design of this effectiveness evaluation, extensively described in the recently published protocol of the study [39], was based on the approach used to assess the CRHT experience in the canton of Aargau [20] and adapted to the regional setting of Canton Ticino. The CRHT intervention, the quantitative study design and the statistical analysis to assess the effectiveness of CRHT in comparison with standard inpatient treatment are described in details here below. All methods were carried out in accordance with relevant guidelines and regulations.

### The intervention

In April 2016 one of the CPC acute wards was closed and replaced by a CRHT team, available 24/7 (on call from 10:30 pm to 7 am), formed of three doctors fully trained in psychiatry (i.e. a full-time consultant psychiatrist, a part-time psychiatrist, and a part-time senior consultant psychiatrist on call), ten mental health nurses, one team manager, a part-time clinical psychologist and a part-time social worker.

Access to the CRHT service was managed by the CPC triage, by Accident and Emergency teams, local community mental health professionals, private psychiatrists and general practitioners. The treatment was administered in daily home visits to the patients, the family members and/or the caregivers, and consisted in a structured psychoeducational approach to acute psychiatric crises aimed at reducing symptoms and preventing future relapses. The number of home visits per day varied based on patients’ needs. The intervention included standard elements of acute care, such as crisis management, pharmacotherapy, psychoeducation, psychotherapy and social care. Though standardization was sought, every intervention was individually tailored according to the specific needs of each patient. The CRHT team’s activities included monitoring of symptoms; adherence to and side effects of pharmacological treatment; identification and management of contextual life-threatening hazards; provision of emotional, social and psychological support to patients, families and/or caregivers; connection with other health and social care services and providers; planning of discharge meetings and follow-ups. The CRHT team also ensured an active collaboration with local services/practitioners to provide long-term support to patients.

The CRHT service was offered to patients aged 18 to 65 who suffered from an acute psychiatric crisis that would have required hospitalization. CRHT was not offered to patients with acute alcohol or drug intoxication, extreme agitation and/or aggressive behaviour, acute risk of suicide/self-harm or representing a significant risk for others. Further CRHT exclusion criteria were compulsory admission, being an inmate and place of residence (patients living in the southern area of Ticino could not be served due to practical reasons).

### Study design, sample and data collected

We used a quasi-experimental design based on existing groups, namely a natural experiment based on geography [40], to allocate patients to the intervention and control groups according to the place of residence. The assumption of casual distribution of socio-demographic and relevant health characteristics across geographic groups was supported by preliminary statistics on patients treated in the acute wards of the northern (*n* = 108) and southern (*n* = 263) areas of the Canton during a period of six month before the introduction of CRHT. Bivariate chi-square and Mann–Whitney tests showed no significant difference in both the socio-demographic (gender, *p*-value = 0.069; age, *p*-value = 0.266; education, *p*-value = 0.404; civil status, *p*-value = 0.404; etc.) and clinical (main psychiatric diagnosis, *p*-value = 0.843; number of previous admissions, *p*-value = 0.527; severity of psychiatric symptoms at admission *p*-value = 0.855; etc.) characteristics. Patients living in the southern area of the Canton were recruited only if theoretically willing to accept CRHT, and could be legitimately allocated to the control group and deemed comparable to those allocated to the intervention group. Under these conditions, according to the assumption of conditional geographic treatment ignorability [40], the assignment to treatment and control groups can be considered to approach randomization when controlling for a set of pre-treatment covariates (gender, age, primary diagnosis, etc.).

The recruitment of patients took place over a two-years period, from mid-March 2017 to the beginning of April 2019. All patients recruited living in the northern area of the Canton were treated with CRHT and included in the intervention group, those from the southern area received care-as-usual (i.e. hospitalization) and were included in the control group. Eligibility for the study was assessed according to the criteria giving access to CRHT (above), with the additional condition of a hospitalization period of at most 48 h before being transferred to the CRHT service. Moreover, certificates of compulsory hospitalization rescinded and/or acute drug or alcohol intoxications resolved within 48 h from hospitalization granted eligibility for the corresponding patients. Finally, patients with a treatment period too short (less than seven days) were further excluded because they most likely did not actually meet the criteria for a major acute psychiatric crisis. The minimal sample size needed to ensure a statistical power of 80% at the 5% significance level for a two-tailed hypothesis test was 142 patients in each study arm, based on previous evidence on mean differences over a 12 to 24 months period of the Health of the Nation Outcome Scales (HoNOS) overall score [19]. HoNOS is a validated 12-items measure, commonly used to assess the health and social functioning of people with severe mental illness [41].

Data were part of routine medical data collection. Clinical data (psychiatric symptoms severity, primary psychiatric diagnosis, etc.) were collected by trained psychiatrists. For both groups we considered data on four main outcomes. During the treatment period, we used the HoNOS total score to quantify the change in the severity of symptoms between admission and discharge, and computed the treatment length. During the 2-years follow-up period, we focused on readmission rates, to either CPC or CRHT, and total treatment length after readmission. We also considered data on patients’ socio-demographic and clinical characteristics, namely gender, age, nationality, educational level, civil status, living arrangement, working condition, primary psychiatric diagnosis, presence of a secondary diagnosis, compulsory admission, number of previous hospitalizations and HoNOS score at the admission. Finally, safety was measured during the treatment period according to the frequency of serious adverse events, defined as suicide or suicide attempts, major self-harm episodes requiring emergency room visits or hospitalizations, acute alcohol/drug intoxications and major aggressions to caregivers or family members.

### Statistical analysis

We compared the main outcomes and the socio-demographic and clinical characteristics of the patients between the intervention and control group using descriptive statistics and χ^2^ and Mann–Whitney tests (for count and continuous variables, respectively), and applied the Monte Carlo method to estimate the Mann–Whitney test *p*-value because of the expected skewness in the distribution of continuous variables (e.g. treatment length).

To improve causal inference, we used propensity scores (PS) matching [42] for the main analysis of differences between the intervention and control group for each outcome. First, we selected the variables to include in the PS reference model using the approach proposed by Cattaneo, Drukker and Holland [43]. We ran a set of independent logistic regression models with intervention/control group membership as dependent variable and all the possible combinations of sociodemographic and clinical variables as predictors, and quantified the goodness-of-fit using the Bayesian Information Criterion (BIC). We selected as reference PS model the one with the best goodness-of-fit (i.e. the lowest BIC). Then, using the socio-demographic and clinical variables retained in the PS reference model, we estimated the Average Treatment Effect (ATE) of CRHT separately on each of the outcomes using the Augmented Inverse Probability Weighting (AIPW). Moreover, we used log-transformation for the strongly skewed outcomes, Weighted Nonlinear Least Squares (WNLS) instead of maximum likelihood and bootstrapped standard errors to provide additional robustness to the estimates. We controlled for the covariates balance between the intervention and control groups according to the overidentification test for covariate balance proposed by Imai and Ratkovic [44]. We addressed missing data using listwise deletion.

We conducted all statistical analyses with Stata/IC 16.0 (StataCorp, 4905 Lakeway Drive, College Station, Texas, USA).

## Results

### Patients’ disposition and safety

**Fig. 1:**
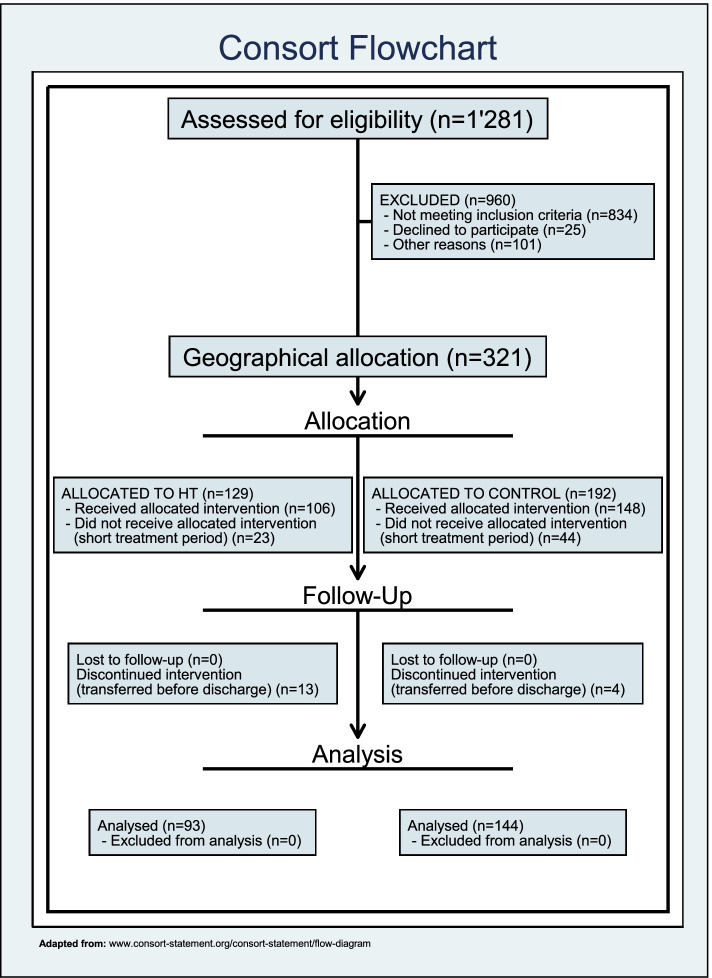
Patients’ disposition (CONSORT Flowchart)

Between mid-March 2017 and the beginning of April 2019, 1′281 patients were referred to the CPC for hospital admission and assessed for study eligibility. 834 patients were excluded because they did not meet the inclusion criteria, while 25 declined participation and 101 were excluded for other reasons (e.g. therapeutic reasons, cognitive problems, lack of suitable living environment, language problems). Figure 1 shows the flowchart of participants, according to the Consolidated Standards of Reporting Trials (CONSORT) recommendations.

Overall, we enrolled 321 patients, 192 in the control group (i.e. hospitalized) and 129 in the intervention group (i.e. CRHT care). Sixty-seven patients (23 in the intervention group and 44 in the control group) were further excluded because the treatment period was too short (less than seven days), while 17 additional patients had to be excluded because they were transferred to another healthcare facility outside the CPC before the end of the treatment (13 in the intervention group and 4 in the control group). A total of 237 patients (93 in the intervention group and 144 in the control group) were finally available for the data analysis. No patient in the intervention group was hospitalized at the CPC before the end of the home treatment, and no serious adverse event was observed during the treatment period in both arms.

It should be noted that, according to the sample size calculation (above), the intervention and control groups were originally planned to be of equal size. However, the recruitment of patients for the intervention group was characterized by a “saturation” effect. Throughout time less and less new patients living in the northern region of the Canton referred for hospital admission resulted eligible for the study and/or accepted to participate, which led to a smaller sample size for the intervention group at the end of the recruitment period.

### Statistical analysis results

Table 1 presents the demographic and clinical characteristics of patients in the intervention and control groups at admission. There were no significant differences between groups by median age (*p*-value = 0.152), citizenship (*p*-value = 0.164), educational level (*p*-value = 0.187) and working status (*p*-value = 0.515). Instead, in the intervention group we found higher percentages of women (*p*-value = 0.001) and of married people (*p*-value = 0.016), while the proportion of patients living alone was significantly higher in the control group (*p*-value = 0.010).

**Table 1:** Demographic and clinical characteristics of intervention and control groups

**Characteristics**	**Intervention group (*n* = 93)**	**Control group (*n* = 144)**	**Statistical test for the difference**^a^
***Demographic***			
Female gender, n(%)	57 (61.3)	55 (38.2)	χ^2^(1) = 12.173***
Age, years: median (IQR^b^)	41.7 (20.3)	45.7 (20.1)	z = 1.432
Swiss citizenship, n(%)	74 (79.6)	103 (71.5)	χ^2^(1) = 1.969
Educational level, n(%)			
*None/compulsory*	40 (43.0)	67 (46.5)	χ^2^(2) = 3.467
*Secondary*	47 (50.5)	59 (41.0)	
*Tertiary*	6 (6.5)	18 (12.5)	
Married, n(%)	34 (36.6)	32 (22.2)	χ^2^(1) = 5.700*
Living alone, n (%)	32 (34.4)	74 (51.4)	χ^2^(1) = 6.665*
Employed, n (%)	18 (19.4)	33 (22.9)	χ^2^(1) = 0.429
***Clinical***			
Compulsory admission, n (%)	15 (16.1)	43 (29.9)	χ^2^(1) = 6.001*
Primary diagnosis (ICD-10), n (%)			
*Mental and behavioural disorders due to use of psychoactive substances (F1)*	4 (4.3)	19 (13.2)	χ^2^(5) = 20.706**
* Schizophrenia, schizotypal and delusional disorders (F2)*	24 (25.8)	45 (31.3)	
* Mood [affective] disorders (F3)*	29 (31.2)	41 (28.5)	
*Neurotic, stress-related and somatoform disorders (F4)*	12 (12.9)	19 (13.2)	
* Disorders of personality and behaviour in adult persons (F6)*	24 (25.8)	14 (9.7)	
* Other disorders (F5, F8, F9, Z)*	0 (0.0)	6 (4.2)	
Presence of a secondary diagnosis, n (%)	59 (63.4)	87 (60.4)	χ^2^(1) = 0.219
Num. previous hospitalizations: median (IQR)	1 (3)	2 (4)	z = 2.887**
HoNOS at admission^c^: median (IQR)	18 (8)	16 (9)	z = -1.045

^a^ χ^2^ test for categorical variables; Fisher’s exact test was used in presence of cells with a count lower than 5. Mann–Whitney test for continuous variables; the 95% confidence intervals for *p*-values used to assess statistical significance were estimated using the Monte Carlo method based on 10′000 samples

^b^ IQR = Interquartile range

^c^ Data on the HoNOS were missing for 6 patients in the intervention group and 12 in the control group

^*^p-value < 0.05, ** *p-value* < 0.01, *** *p-value* < 0.001

Participants in the intervention group had on average significantly lower mental and behavioural disorders due to the use of psychoactive substances (F1; *p*-value = 0.024) and significantly higher personality and behaviour disorders in adult persons (F6; *p*-value = 0.001). Moreover, the intervention group counted fewer certificates of compulsory hospitalization rescinded before recruitment (*p*-value = 0.016) and a lower median number of previous hospitalizations at the clinic (*p*-value = 0.004). No significant differences were found in the presence of a secondary diagnosis (*p*-value = 0.640) and in the median HoNOS score at admission (*p*-value = 0.296).

Table 2 shows the outcomes for the intervention and control groups. While the median reduction in the HoNOS total score at discharge resulted homogeneous between the two groups (*p*-value = 0.731), the median treatment length was higher for the intervention group (*p*-value = 0.001). Irrespective of the group considered, around half of the patients were readmitted to the CPC or to CRHT at least once within two years from discharge (*p*-value = 0.772). Despite the lower median in the intervention group, also the total treatment length related to single or multiple readmissions did not differ significantly between the two groups (*p*-value = 0.800).

**Table 2:** Outcomes for the intervention and control groups

**Outcomes**	**Intervention group (*n* = 93)**	**Control group (*n* = 144)**	**Statistical test for the difference**^a^
HoNOS difference at discharge^b^: median (IQR^c^)	-8 (8)	-8 (8)	z = -0.345
Treatment length: median (IQR)	36 (23.5)	27 (17)	z = -3.251**
Readmission within 2 years^d^, n(%)	46 (49.5)	74 (51.4)	χ^2^ (1) = 0.084
Number of readmission days^e^: median (IQR)	41 (56.8)	52 (62.5)	z = 0.254

^a^ χ^2^ test for categorical variables. Mann–Whitney test for continuous variables; the 95% confidence intervals for *p*-values used to assess statistical significance were estimated using the Monte Carlo method based on 10′000 samples

^b^ The HoNOS difference at discharge corresponds to the difference between the total HoNOS score at discharge and the total HoNOS score at admission. Data were missing for 8 patients in the intervention group and 21 in the control group

^c^ IQR = Interquartile range

^d^ Readmission within 2 years indicates if a patient was readmitted to the CPC and/or to CRHT at least once during the 2 years period following discharge

^e^ The number of readmission days corresponds to the total number of inpatient/CRHT days related to single or multiple readmissions during the 2 years period following discharge. Data were available for 39 patients in the intervention group and 74 in the control group

^*^p-value < 0.05, ** *p-value* < 0.01, *** *p-value* < 0.001

Table 3 displays the PS model selected according to the approach proposed by Cattaneo, Drukker and Holland [43]. The variables important for balancing the two groups were gender, living arrangement, compulsory admission, diagnosis of personality and behaviour disorders in adult persons (F6) and number of previous hospitalizations; the latter was included in the model in spite of not being statistically significant at the 5% level. Predicted probabilities, ranging on average from 0.337 to 0.663, were sufficiently far from the extreme values of 0 and 1 to suggest no particular concern about the selected model.

**Table 3:** Propensity Scores model selected

Covariates		Dependent variable: Intervention/control group membership	
		**Coefficient (SE**^a^**)**	**95% confidence interval**
Female gender		0.837**	(0.271;1.403)
		(0.289)	
Living alone		-0.623*	(-1.215;-0.032)
		(0.302)	
Compulsory admission		-0.721*	(-1.413;-0.029)
		(0.353)	
Disorders of personality and behaviour in adults (F6)		1.338**	(0.561;2.115)
		(0.397)	
Number of previous hospitalizations		-0.028	(-0.077;0.022)
		(0.025)	
Constant		-0.557*	(-1.082;-0.032)
		(0.268)	
Number of observations (n)		237	
LR χ^2^ (5)		34.59***	
Bayesian Information Criterion (BIC)		315.707	
Predicted probabilities^b^			
CPC^c^	Pr^d^(CPC): mean (SD^e^)	0.663 (0.156)	
	Pr(CRHT): mean (SD)	0.337 (0.156)	
CRHT^f^	Pr(CPC): mean (SD)	0.521 (0.188)	
	Pr(CRHT): mean (SD)	0.479 (0.188)	

^a^*SE* Standard Error

^b^ Predicted probabilities for each treatment level were computed and summarized conditional to each treatment level

^c^*CPC* Cantonal Psychiatric Clinic

^d^*Pr* Probability

^e^*SD* Standard Deviation

^f^*CRHT* Crisis Resolution Home Treatment

^*^p-value < 0.05, ** *p-value* < 0.01, *** *p-value* < 0.001

Table 4 presents the estimates of the ATE of CRHT on the outcomes. The overidentification test for covariate balance showed that the selected PS model successfully balanced the intervention and control groups (*p*-values of the test of 0.334 or higher for all models). We found no significant ATE of CRHT on the HoNOS score difference at discharge (*p*-value = 0.315), on readmission rates within two years from discharge (*p*-value = 0.563) and on the number of readmission days for patients readmitted at least once within two years from discharge (*p*-value = 0.771). The ATE on the treatment length was instead statistically significant; the duration of the treatment in the intervention group was higher (*p*-value = 0.002).

**Table 4:** Estimates of the ATE of CRHT

**AIPW**^a^**PS**^**b**^** models** ***1) linear outcome model*** ***2)* + *4) log-linear outcome model*** ***3) logistic outcome model***		**Coefficient (Bootstrap SE**^c^**)**	**95% confidence interval**
1) HoNOS difference at discharge^d^	ATE^e^ (CRHT^f^ vs CPC^g^)	1.026	(-0.791;2.842)
(*n* = 208)		(0.927)	
	CPC	-9.434***	(-10.706;-8.163)
		(0.649)	
2) ln^h^(Treatment length)	ATE (CRHT vs CPC)	0.296**	(0.088;0.504)
(*n* = 237)		(0.106)	
	CPC	3.182***	(3.046;3.319)
		(0.070)	
3) Readmission within 2 years^i^	ATE (CRHT vs CPC)	0.039	(-0.093;0.170)
(*n* = 237)		(0.067)	
	CPC	0.493***	(0.408;0.577)
		(0.067)	
4) ln(Number of readmission days^j^)	ATE (CRHT vs CPC)	-0.071	(-0.460;0.319)
(*n* = 120)		(0.199)	
	CPC	3.833***	(3.580;4.085)
		(0.129)	

^a^*AIPW* Augmented Inversed Probability Weighting

^b^*PS* Propensity Scores

^c^*Bootstrap SE* Bootstrapped Standard Errors: they were obtained through 1′000 replications

^d^ The HoNOS difference at discharge corresponds to the difference between the total HoNOS score at discharge and the total HoNOS score at admission

^e^*ATE* Average Treatment Effect

^f^*CRHT* Crisis Resolution Home Treatment

^g^*CPC* Cantonal Psychiatric Clinic

^h^*ln* Natural logarithm

^i^ Readmission within 2 years indicates if a patient was readmitted to the CPC and/or to CRHT at least once during the 2 years period following discharge

^j^ The number of readmission days corresponds to the total number of inpatient/CRHT days related to single or multiple readmissions during the 2 years period following discharge

^*^p-value < 0.05, ** *p-value* < 0.01, *** *p-value* < 0.001

## Discussion

The findings of our study show that CRHT could actually be an effective substitute of care-as-usual for patients suffering from an acute mental crisis that would require hospitalization.

The psychiatric symptoms largely improved at discharge without differences between the intervention and control groups (median reduction of eight points in the HoNOS score for both groups), indicating a very similar effectiveness of the two treatment options. This result is perfectly in line with several previous findings [12, 19–21, 24, 26], thus reinforcing the empirical evidence suggesting that CRHT and care-as-usual are equally effective in reducing psychiatric symptoms of patients suffering from acute crises.

The treatment has proven to be significantly longer in the intervention group, with a median treatment length of 36 days against 27 in the control group (after PS matching + 29.6% on average, *p*-value = 0.002). This result is in line with a recent Swiss study [26], but in contrast with two others that found no significant difference between CRHT and care-as-usual treatment length [20, 25]. It should be noted that the median duration of CRHT in Ticino lowered over time from 37 days in 2016 to 29 days in 2020. Since in our study patients were mainly recruited in 2017 and 2018, a higher CRHT duration could be related to the lack of experience in the new clinical setting, and this difference might decline over time due to a “learning effect”. Moreover, additional bivariate analysis showed that a higher CRHT duration might be related to some patients’ characteristics. For example, a higher median CRHT duration was related to the fact of not living alone (CRHT: 41 days vs CPC: 26 days; *p*-value = 0.011). More in general, treatment length might be related to the complexity of the cases considered [45]. Further research is therefore needed to assess under which conditions CRHT and care-as-usual treatment lengths are actually comparable.

In both the intervention and the control groups, 50% of the patients were readmitted to the CPC and/or to CRHT at least once during the two years following discharge; the treatment length related to these readmissions did not differ significantly between the two groups. The finding concerning the readmission rate is in line with several studies [17, 20, 21, 26] but in contrast with some others reporting lower readmission rates for CRHT patients [7, 9, 10, 12, 19, 24, 25], while the result regarding the treatment length of readmissions is line with a recent Swiss study [26] but in contrast with the majority of previous findings [10, 16, 17, 19, 20, 23, 25]. Such differences might be due to several factors of heterogeneity across studies, as for example the type of patients considered (e.g. some studies included also patients without an immediate need of hospitalization [7, 19]), the aim of CRHT (e.g. reduce hospital days by rapid and facilitated discharge instead of focusing on readmissions prevention [20]), or any other element that could influence the long-term effect of the treatment after discharge. Further detailed studies are needed to investigate the effect of different CRHT regimes on readmission rates and lengths.

The presence of more married women with affective disorders in the intervention group is in line with other recent findings [26, 34, 46–51], and raises the question of the impact of CRHT on the entire mental healthcare system. The combination of this finding with the longer CRHT treatment duration might imply the risk of a redistribution of resources towards a specific group of CRHT patients. However, this effect is not confirmed by the post-study observational evidence indicating a lowering of CRHT duration throughout time. But the significant difference in the type of patients treated in the CRHT and hospital settings highlights the risk of creating different treatment environments, considering that the most complex cases (i.e. aggressive patients, patients at risk of suicide or self-harm, compulsory admissions and patients with acute intoxications) are normally excluded from CRHT. This may have consequences on the mix of patients in the inpatient wards. The concentration of complex cases in the psychiatric hospital could worsen the atmosphere of care, and could therefore have a negative impact on the working staff (also in terms of recruitment on the labour market) and on the type of specific therapies offered. Further research is therefore needed to understand in deeper details the impact of the introduction of the CRHT service on the entire mental healthcare system.

This study is characterized by some limitations. The first was the impossibility of randomizing the recruited patients because of logistic and ethical problems; we could overcome this limitation, at least approximately, by using the natural experiment design based on geography that allowed approaching real randomisation. The second was the “saturation” effect related to patient’s recruitment for the intervention group, which prevented from having similar sample sizes for both groups and may have increased the chances of Type II errors (i.e. the fact of incorrectly assessing the non-significance of a difference between the two groups). However, with the exception of the statistically significant difference in the treatment length, the comparison between the two groups showed a high degree of homogeneity in the outcomes, suggesting a good reliability of the obtained statistical results. Third, the study was conducted in a single psychiatric hospital, which may prevent the generalizability of findings to other settings.

## Conclusions

The study confirmed the effectiveness of CRHT as a substitute of hospitalization for patients suffering from acute psychiatric crises. Our results showed the comparability between CRHT and inpatient treatment outcome in terms of psychiatric symptoms reduction at discharge, findings that are robustly supported in the literature. However, a longer treatment for CRHT patients and similar readmission rates and readmission treatment lengths do not find unanimous support in the literature. This is probably due to heterogeneity across settings, since CRHT is a very complex intervention where details matter and these aspects may differ across studies (e.g. geographic setting, aim of CRHT, patients considered, team composition, etc.). In this sense, additional studies are needed in order to understand in deeper details the effects of the various characteristics of CRHT on the treatment outcomes. Recent contributions investigating the characteristics of patients who benefited the most from CRHT [46] and of patients relapsing after CRHT [52] pave the way for future research in this direction.

## Data Availability

The datasets used and/or analyzed during the current study are available from the Cantonal Psychiatric Clinic (contact: Maddalena Alippi) on reasonable request.

## Abbreviations

CRHT: Crisis Resolution Home Treatment
RCT: Randomised Controlled Trial
CPC: Cantonal Psychiatric Clinic
ITT: Intention-to-treat
CTGI: Conditional Geographic Treatment Ignorability
HoNOS: Health of the Nation Outcome Scales
PS: Propensity scores
BIC: Bayesian Information Criterion
ATE: Average Treatment Effect
AIPW: Augmented Inverse Probability Weighting
WNLS: Weighted Nonlinear Least Squares
CONSORT: Consolidated Standards of Reporting Trials
